# The Correlation Between Wearing Face Masks and Skin Damage in Adults During the COVID-19 Pandemic: A Cross-Sectional Study in Jeddah, Saudi Arabia

**DOI:** 10.7759/cureus.31521

**Published:** 2022-11-15

**Authors:** Mohammed Abduljabbar, Duha E Kalthoum, Marwan Bakarman, Iman Wahby Salem, Zakeiah Alsulaimani, Wedyan Alharbi, Shahad Shawish, Rahaf Alsobhi

**Affiliations:** 1 Dermatology, King Abdulaziz University Hospital, Jeddah, SAU; 2 Community Medicine, King Abdulaziz University, Rabigh, SAU; 3 Medicine, King Abdulaziz University, Rabigh, SAU; 4 Community Medicine, King Abdulaziz University, Jeddah, SAU

**Keywords:** covid-19, eczema, rash, acne, surgical mask, n95, face mask

## Abstract

Background

The impact of COVID-19 on the world is rapidly spreading among countries. According to WHO, wearing face masks was recommended to prevent its spread. After regular use of face masks, some people have experienced common skin disorders such as facial acne, rash, and eczema. This paper aims to cite the prevalence and potential risk of wearing a face mask on the skin by exploring some of the rationales that have been established in the literature.

Methodology

A cross-sectional study was carried out in Jeddah, Saudi Arabia in November 2021. A self-administered online questionnaire was distributed among 389 participants from the adult public to find the correlation between face mask wearing and skin damage during the COVID-19 pandemic. Statistical analysis was conducted using the IBM SPSS statics for windows, version 25.0 (IBM Corp., Armonk, USA) to evaluate and test the hypothesis.

Results

The study included 389 participants; 63.8% of them were female and 36.2% were male. The main result of this study was that there was a statistically significant association between the duration and frequency of wearing a face mask and developing skin damage. 58.1% of the participants were using face masks for more than 4 hours. Furthermore, 22% and 59.1% of the male and female participants, respectively, said they noticed adverse skin reactions on their faces after using a face mask.

Conclusion

Our study revealed that 46% of the participants noticed adverse skin reactions on the face by wearing a face mask. Females had a significantly higher chance of developing skin irritation than males.

## Introduction

Coronavirus disease 2019, known as COVID-19, is an infectious disease caused by a new virus related to the coronavirus family that causes severe acute respiratory syndrome (SARS) [1]. It is transmitted through respiratory droplets when infected individuals cough or sneeze, where the droplet can travel up to one meter, affecting those around. Furthermore, if the infected person is not careful, by touching their nose or mouth, for example, they can further spread the virus when other people touch the surfaces touched by them [2]. While most people who contract the disease, experience mild to moderate respiratory problems, many other cases have been severe and even life-threatening. The virus took the world by storm, spreading fast across country borders to continents from its origin in Wuhan, China, all the way to the Americas, prompting countries to impose strict public health measures to protect their citizens. According to the World Health Organization (WHO), mandating the face mask is one of the most important preventive measures to stop the transmission of disease between people [3].

Wearing a face mask was recommended, and in some countries imposed, to reduce the spread of infectious agents by covering the mouth and the nose [4-5]. Masks can either be disposable, such as surgical masks and N95 respirators, or reusable woven cloth. It was crucial to use a face mask throughout the COVID-19 pandemic to reduce the transmission of droplets from the users’ mouths and noses [6]. As of December 2020, there were over 79.2 million cases reported since the start of the COVID-19 pandemic and 362,066 cases in Saudi Arabia alone [7]. Therefore, Saudi Arabia imposed strict public health measures to control the spread of COVID-19 and protect its citizens. Some of these measures included curfews, city lockdowns, shutting air and land travel, and wearing face masks in public places as well as in the company of other people [6].

While there are no known global statistics to indicate the prevalence of face-mask wearing, 88% of the world’s population live in countries that mandated wearing face masks in public and in gatherings [8]. Furthermore, global market predictions indicated that face mask sales were expected to grow exponentially by over 400% in 2021 compared to 2019 [9]. In Saudi Arabia, health authorities imposed a financial fine on those that did not wear masks out in public and in gatherings with other people [10]. Studies showed that 87.2% of people in Saudi adhere to face mask regulations in public places, 80.5% in workplaces, and 47.5% in social gatherings [11].

While wearing face masks helps against the COVID-19 spread, some adverse skin reactions have been reported among people after the regular use of face masks [4]. A study in Wuhan in 2019, found that the most common skin damages were dryness (68.6%), erythema (60.4%), and maceration (52.9%) [12]. Another study conducted at Khon Kaen University, Thailand [4] showed a 54% prevalence of skin reaction cases due to wearing face masks, mainly acne (40%), face eczema (18%), and urticaria in the face (16%). The study concluded that these symptoms are more apparent among those who frequently wear a disposable surgical mask, compared to those who wear a cloth mask that is made of washable fabric and fitted around the mouth and nose [4].

There is a shortage of reports on the true association and impact of face masks and skin damage worldwide. Therefore, this paper aims to cite the prevalence and potential risk of wearing face mask on the skin.

## Materials and methods

This is a cross-sectional study conducted to find the correlation between wearing face masks and skin damage during the COVID-19 pandemic in Jeddah, Saudi Arabia. The study included adults males and females, aged 18 and above, residents and locals living in Jeddah. The study excluded people who did not live in Jeddah city and pregnant women. The study was approved by the Institutional Review Board of King Abdulaziz University (reference No 499-21). 

A self-administered online questionnaire in Arabic was used from November 2nd until the 23rd of 2021. The study used two social media platforms, WhatsApp and Snapchat, to distribute the questionnaire to contacts and continued to snowball to reach more participants. Initially, 502 participants completed the survey. After excluding participants who did not fit the inclusion criteria, the sample size reached n= 389 participants, which met our target sample that we calculated using Raosoft (2004) Sample Size Calculator (Raosoft, Inc., Seattle, USA) based on the population size. Among the 389 participants, 141 male (36.2%) and 248 female (63.8%) participants were enrolled, introduced to the study, and taken consent from. 

This survey was a self-administered questionnaire which was used in previous studies [4-13] and translated and validated by three experts (two epidemiologists and one dermatologist). The questionnaire was divided into two sections: the first section was a collection of multiple choice questions regarding demographics (age, gender, marital status, place of residence, education level, and occupation); the second part contained questions regarding personal face mask wearing routine (type of face mask and amount of time wearing it) and the skin condition before and after wearing the face mask, such as acne, erythema (defined as skin redness around the area covered in the face mask), pigmentations (defined as skin discoloration in the area covered in the face mask), itching, rash, or others. 

Statistical analysis was conducted using SPSS Statistics version 25 (IBM, Armonk, USA) to evaluate and test the hypothesis. Descriptive statics of frequencies and percentages were used to analyze the demographics. In addition, a univariate binomial logistic regression was used to test the predictors of the binary outcome variable. Chi-square was used to test and describe the relation between two categorized variables, with 95% Confidence Interval (CI) and p<0.05 used as the cut-off value for significance.

## Results

A total of 389 participants were included in this study. A description of socio-demographics, face mask behavior, and skin changes are shown in Table 1. 63.8% of the respondents were females and 36.2% were males. Most participants were between the ages of 18 to 24 years (39.8%), and the majority had a higher educational level, i.e., universities and postgraduates (82%). 76.3% of the participants were non-healthcare workers (n=297). 83.5% of the participants reported using surgical masks, 19.3% wore cloth masks, 7.5% wore surgical masks with cloth covering, and only 2.8% reported using N95 masks. When participants were asked if they noticed any skin changes in the face area covered by the mask, only 46% of participants said yes, while 56% said no. 46% of participants said they wore the face masks four to eight hours, 42% said they keep it on for four hours, and only 12% said they keep it on for longer than eight hours. When asked about how often they changed their face mask, 44% of participants said they changed it every day, 12% said every eight hours, 10% said every four hours, and only 5% said they change their face mask every 12 hours.

**Table 1:** Characteristics of study participants, n=389

Characteristics		Frequency (n)	Percentage (%)
Gender	Male	141	36.2
	Female	248	63.8
Age (years)	18-24	155	39.8
	25-34	81	20.8
	35-44	71	18.3
	45-54	41	10.5
	>55	41	10.5
Education	High school or less	70	18.0
	University/postgraduate	319	82.0
Occupation	Non-healthcare workers	297	76.3
	Healthcare workers	92	23.7
History of allergy	No	338	86.9
	yes	51	13.1
Type of face mask	Surgical mask	325	83.5
	Cloth mask	75	19.3
	Surgical mask with cloth covering	29	7.5
	N95 mask	11	2.8
Did you notice adverse skin reaction on the face covering by face mask?	No	210	54.0
	Yes	179	46.0
Time of face mask wearing (hours)	<4	163	41.9
	4-8	179	46.0
	>8	47	12.1
Frequency of face mask changing (hours)	Every 4	41	10.5
	Every 8	47	12.1
	Every 12	20	5.1
	Everyday	171	44.0
	After a day	110	28.3

**Figure 1:**
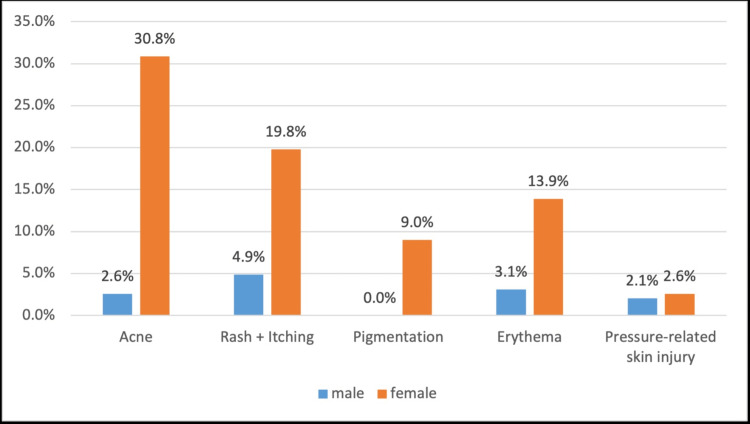
The prevalence of face mask adverse skin reaction by gender, n=389 Adverse skin reaction across gender for Acne X2 (2) = 68, p<0.001; Rash + Itching X2 (2) = 15, p<0.001; Pigmentation X2 (2) = 22, p<0.001; Erythema X2 (2) = 11, p<0.001; Pressure-related injury X2 (2) = 0.6, p>0.001.

Figure 1 shows the prevalence of face mask adverse skin reactions by gender. Compared to males, more females reported developing adverse skin reactions to wearing a face mask. Out of the participants who noticed acne formation after wearing a face mask, 30.8% were females and only 2.6% were males. 19.8% of participants who developed rash and/or itching were females and only 4.9% were males. 13.9% of participants who reported developing erythema were females and 3.1% were males. 9% of all participants who developed pigmentations were females. 2.6% of those who reported pressure-related skin injury were females and 2.1% were males. All these differences were statistically significant (p<0.001) except for pressure-related injuries (p=0.459).

**Figure 2:**
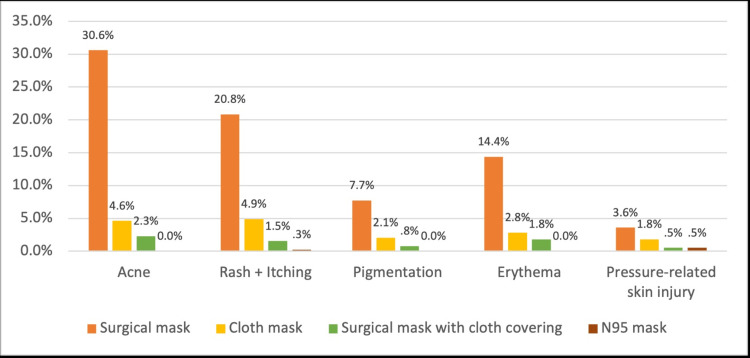
Face mask adverse skin reactions and their prevalence by face mask type Adverse skin reaction against face mask type for Acne X2 (4) = 16, p=0.05; Rash + Itching X2 (4) = 9, p=0.4; Pigmentation X2 (4) = 6, p=0.7; Erythema X2 (4) = 9, p=0.4; Pressure-related injury X2 (4) = 30, p<0.001.

Figure 2 shows the prevalence of face mask adverse skin reactions by type of face mask. Most participants who reported developing acne wore surgical masks (30.6%) while only 4.6% of participants who wore cloth masks developed acne and 2.3% of those who wore surgical masks with cloth covering developed acne. 20.8% of participants who reported developing rash and itching wore surgical masks, 4.9% wore cloth masks, and 1.5% wore surgical masks with cloth covering. Of the participants who said they developed pigmentation, 7.7% wore surgical masks, 2.1% wore cloth masks and only 0.8% wore surgical masks with cloth covering. Of those who reported developing erythema, 14.4% wore surgical masks, 2.8% wore cloth masks and 1.8% wore surgical masks with cloth covering. Among participants who reported developing pressure-related skin injury, 3.6% wore surgical masks, 1.8% wore cloth masks, 0.5% wore surgical masks with cloth covering and 0.5% wore N95. Except for pressure-related injury, which yielded a statically significant result (p<0.001) all these differences were statistically insignificant (p-value for acne = 0.05, p-value for rash + itching = 0.4, p-value for pigmentation = 0.7, and p-value for erythema = 0.4).

Table 2 examines the risk of adverse skin reactions among some of the variables as predictors for the outcome. Females are five times more likely to report developing adverse skin reactions compared to males (95% CI 3.3-8.4, p=0.00 <0.05). Similarly, healthcare workers are 4.4 times more likely to report developing adverse skin reactions than non-healthcare workers (95% CI 2.6-7.4, p=0.00 <0.05). Regarding the type of face mask, participants who reported wearing surgical face masks were 1.9 times as likely to report adverse skin effects as opposed to those who don’t wear them (95% CI 1.1-3.4, p=0.02 <0.05). Furthermore, there was no significant difference in contribution to adverse skin reactions in participants who reported wearing cloth masks versus those who did not wear them (95% CI 0.52-2.4, p=0.51 >0.05). And no statistically significant difference was found among those who reported wearing the N95 mask when compared to those who did not (95% CI 0.05-1.2, p=0.08 >0.05). For the average duration of wearing the face mask per day, participants who wore it for longer than four hours, specifically 4-8 hours, were more likely to report adverse skin reactions than those who did so for less than four hours (OR 2.7 (95% CI 1.7-4.2, p=0.00 <0.05)) and >8hrs (OR 2.36 (95% CI 1.2-4.6, p=0.01 <0.05)). Finally, participants who took more than one day to change their face mask had a statistically significant lower risk of reporting adverse skin reactions when compared to participants who change their mask every four hours (OR 0.29 (95% CI 0.14-0.6, p=0.00 <0.05)). Yet the duration of consecutive hours for which these same participants keep their masks on is unknown. No statistical significance was found when we compared participants who changed their face mask every 8 hours and every 12 hours (95% CI 0.35-1.9, p=0.61 >0.05 and 95% CI 0.19-1.7p= 0.32 >0.05, respectively).

**Table 2:** Risk estimation of adverse skin reactions using multiple binary logistic regression § Numbers are approximated to the nearest 10th; * Reference category where odds ratio is defined as 1; ^‡ ^Reference category for those who answered 'no' to wearing the type of mask in question, defined as 1; ​​​​​​​^† ^Statistical significance p< 0.05.

Parameters		Odds Ratio (95% CI)^ §^	p-value^†^
Gender	male^*^	-	
	Female	5.3 (3.3-8.4)	0.00
Occupation	Non-healthcare worker^*^	-	
	Healthcare worker	4.43 (2.6-7.4)	0.00
Face Mask type^‡^	Surgical Mask	1.94 (1.1-3.4)	0.02
	Cloth Mask	1.1(0.52-2.4)	0.51
	N95	0.25(0.05-1.2)	0.08
Average duration of wearing the face mask	<4 hrs ^*^	-	
	4-8 hrs	2.7(1.7-4.2)	0.00
	>8 hrs	2.36(1.2-4.6)	0.01
Frequency of changing the face mask	Every 4 hrs^*^	-	
	Every 8 hrs	0.81(0.35-1.9)	0.61
	Every 12 hrs	0.58(0.19-1.7)	0.32
	Everyday	0.77(0.4-1.5)	0.45
	>1 day	0.29(0.14-0.6)	0.00

## Discussion

The aim of this paper is to examine the frequency of adverse skin reactions during the face mask mandate during the COVID-19 pandemic, as well as determine a correlation between skin disorders and face mask practices. This present study included both healthcare workers as well as non-healthcare workers to understand the extent of the effect of face masks on a wider segment of the population [14]. Most previous studies with similar aims have been conducted exclusively among healthcare workers [12,13, 15-19].

Two key findings in this study are that the profession and duration of wearing the face mask had a statistically significant impact on developing adverse skin reactions, and healthcare workers had significantly higher odds (4.4) of reporting adverse skin reactions to wearing face masks compared to non-healthcare workers. Additionally, participants who reported wearing the face mask continuously for longer than four hours had significantly higher odds (2.7) of reporting adverse skin reactions than those who wore it for less than four hours. These findings are consistent with another study conducted in Thailand [4] that demonstrated the effect of the face mask on the skin among 833 participants, of which 43% were healthcare workers and 57% were non-healthcare workers. The results of the aforementioned study showed that healthcare workers had a higher rate of skin reactions than non-healthcare workers (OR (95%CI) = 1.39 (1.05-1.83), p= .021). These results are expected due to the job demands of healthcare workers to always maintain protective equipment on, specifically when dealing with patients who suffer from infectious diseases. The event of skin disorders after wearing face masks can be attributed to the changes in the skin pH, temperature and humidity levels that lead the sebaceous gland secretions to increase under the area covered with face masks for long durations [20]. However, perhaps further analysis to understand healthcare workers’ hygiene practices needs to be investigated in the hope to mitigate any negative effects of wearing face masks for long hours.

Furthermore, this study found that the surgical face mask was the most popular choice of mask, and more adverse skin reactions were reported among those who wore surgical face masks compared to other types of face masks, a finding that is consistent with another cross-sectional study conducted in Denmark in 2019, although the study was focused on healthcare workers only [13]. This is possibly attributed to more widespread use of surgical masks than any other type of face mask in both medical and non-medical settings [4,14]. Among those who wore surgical face masks, acne was the most self-reported skin disorder, followed by rash and itching, erythema, and skin pigmentation. While these findings are consistent with similar studies in the literature [4,15-16], other studies reported itching only when wearing the N95 as a common form of skin irritation around the area of the mask [17,18].

Additionally, females had a significantly higher chance of reporting all kinds of skin irritation than males. A similar gender-specific finding was reported by Skiveren et al - the authors indicated that women in different age groups experienced more face mask-related skin itch than men [13]. Finally, our analysis indicated that participants who take more than a day to change their face mask had less risk of reporting any skin symptoms than those who change their face mask more frequently, an oddity that can be attributed to their individual characteristics and frequency of having to keep the face mask on as opposed to their level of use, like working in one place or using a washable mask. This is certainly an area worth further investigative effort to understand the culpability of face masks in causing skin damage. 

This study has some limitations. First, the previous history of skin disorders and the complete medical history of other medical conditions that influence the skin of participants were not investigated as it was not possible with online surveys. Additionally, since data were collected via self-reported questionnaires, the reported skin disorders were not validated by a medical doctor. Third, results may have been affected by the selection of our sampling technique as the first group of the snowball sample can produce community bias. Finally, caution is advised when interpreting the results of cross-sectional data as they do not demonstrate causality. Reliance on a single point in time measure does not reflect physical changes or monitor disease progression or the incidence of new cases over time.

## Conclusions

Wearing face masks has contributed to the increase in skin damage in adults during COVID-19. Our study shows that the prevalence of adverse skin reactions related to face masks was 46%. And most of them developed acne in the area covered by the face mask. The majority of those who said they had acne wore surgical face masks. Compared to non-healthcare workers, healthcare workers had a greater risk. Wearing a face mask for more than four hours per day was one of the risk variables. Using a cloth mask was determined to have the lowest risk of adverse skin reactions. The findings of this study indicate that instead of a surgical mask, people who are prone to developing skin irritation, particularly non-healthcare workers, should opt to wear a different type of face mask.

There is a dire need to further untangle the relationship between wearing face masks and skin disorders through research. In the meantime, face mask wearers must practice better self-hygiene in terms of washing their hands before touching their face, changing the mask every few hours, properly washing cloth masks before reusing them, and choosing organic fabric with natural dye materials. The public must be educated on the different types of face masks available and how they can choose the one suitable for them.
